# Tomographic Proximity Imaging Using Conductive Sheet for Object Tracking †

**DOI:** 10.3390/s21082736

**Published:** 2021-04-13

**Authors:** Zehao Li, Shunsuke Yoshimoto, Akio Yamamoto

**Affiliations:** 1 School of Engineering, The University of Tokyo, Tokyo 113-8656, Japan; lizehao@aml.t.u-tokyo.ac.jp; 2 Graduate School of Frontier Sciences, The University of Tokyo, Chiba 277-8563, Japan; akio@k.u-tokyo.ac.jp

**Keywords:** capacitive sensing, motion capture, proximity imaging, impedance tomography

## Abstract

This paper proposes a proximity imaging sensor based on a tomographic approach with a low-cost conductive sheet. Particularly, by defining capacitance density, physical proximity information is transformed into electric potential. A novel theoretical model is developed to solve the capacitance density problem using the tomographic approach. Additionally, a prototype is built and tested based on the model, and the system solves an inverse problem for imaging the capacitance density change that indicates the object’s proximity change. In the evaluation test, the prototype reaches an error rate of 10.0–15.8% in horizontal localization at different heights. Finally, a hand-tracking demonstration is carried out, where a position difference of 33.8–46.7 mm between the proposed sensor and depth camera is achieved at 30 fps.

## 1. Introduction

Object tracking technique is crucial in robotics like teleoperation, input interface, and human–robot interaction. Camera-based approaches are commonly adopted [1] when tracking an object at a larger range. Thanks to the recent advancement of machine learning technologies [2,3], camera-based object tracking has been widely used in robot–human cooperation [4] and autonomous driving vehicles [5].

However, at a closer range, visual solutions suffer from restraint of field of view and blocking effect with nontransparent objects. These effects limit the potential of applying visual solutions to close-range proximity sensing. Close-range proximity sensing is necessary for human–computer interfaces such as augmented reality (AR) devices [6]. These devices usually possess limited space for sensors, therefore requiring a thinner proximity sensor for a wider detection area.

Several researches have been carried out regarding close range proximity sensors, and three common approaches are adopted: optical, inductive, and capacitive approaches. Sensors such as Light Detection and Ranging (lidar) or depth sensor are precise in the optical approach [7], but they are hard to implement and still suffer from object occlusion. The inductive approach can realize robust sensors, but it only works with conductive objects [8]. On the other hand, sensors based on the capacitive approach can detect both conductive and non-conductive objects, but they are vulnerable to disturbances and contamination [9,10]. There are also researches that combine inductive and capacitive approaches together [11]. These approaches focus on single proximity sensing, which could only give proximity on one point.

For 3D object tracking, capacitance sensors are often utilized in the form of arrays to generate 3D proximity information. These sensors usually are thin and can be hidden behind some surfaces, and are also not restrained by field of view. Zhang et al. [12] applied a conductive paint pattern onto a wall to track human poses. Ye et al. [13] proposed a high-performance capacitance sensor array by introducing a high sensitivity capacitance measuring circuit and grounded shields around each sensor. However, the sensor array method usually requires complex design of hardware and manufacturing, making it difficult to integrate into arbitrary-shaped devices.

Since the works in [14,15], tomographic approaches have been utilized to determine changes of conductivity across materials through boundary electrodes’ information, which is usually concerned with the pressure distribution on the material. The application of electric impedance tomography sensors has proven its advantage of scalability, versatility, and ease of fabrication. In the previous researches, most of the researches have been focusing on force and pressure imaging [16,17,18]. Some attempts have been made to utilize tomography to image proximity of cylindrical objects; however, it only reveals information on one dimension [19,20]. There have not been endeavor towards using tomographic approach to solve proximity imaging on 2D surfaces.

The purpose of this paper is to capture an object’s 3D position using a thin conductive sheet. Therefore, a novel theoretical model is proposed to find capacitance density, which is related to proximity distribution, from the boundary electrodes on a conductive sheet. Proximity distribution is then used to predict the object’s position. To accomplish the model, the capacitance density on the conductive sheet due to surrounding objects is defined and introduced into the differential equation for the tomographic approach. The system solves an inverse problem to reconstruct the capacitance density, thus estimating the proximity distribution. As the detector is a homogeneous thin layer of a conductive sheet, the detection area can easily be scaled to a larger surface. Additionally, with its hardware architecture, it is possible to incorporate pressure sensing into the same sensor, granting the sensor the abilities of imaging touch interaction and 3D object tracking with low cost and easy manufacturing.

In our previous conference paper, the theoretical model for estimating capacitance density distribution was presented [21]. In this paper, the proximity mapping from capacitance density inside the model is improved. Based on the proposed model, we made a functioning prototype and evaluated the performance of the proposed system. The main contributions of this paper are as follows:

- To present an improved novel proximity imaging method [21] for an object tracking application.
- To develop a proximity imaging sensor using a low-cost conductive sheet and evaluate its proximity and horizontal position estimation accuracy.
- To implement a hand-tracking demonstration as a potential application of the proposed system.

## 2. Methods

### 2.1. Overview

**Figure 1:**
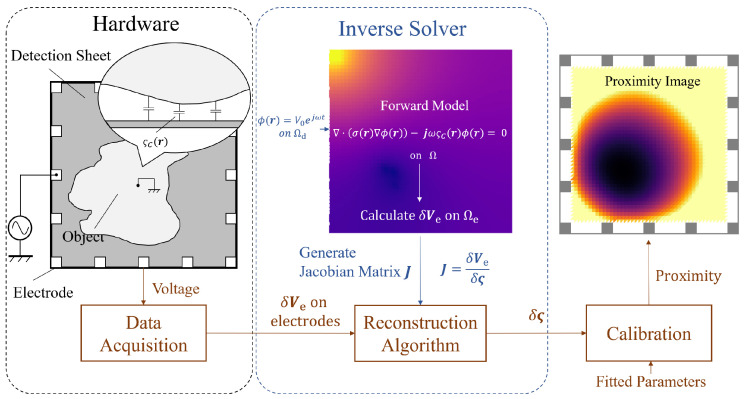
Overview of the proximity imaging. The system interprets physical distance information as voltage data on electrodes and then uses an inverse problem solver to map capacitance density (modified from the work in [21]).

Our final goal is to visualize a proximity distribution on a surface (see Figure 1). First, proximity–capacitance coupling is introduced. A single layer of conductive sheet is used to convert the proximity information to potential on each electrode at the sheet’s boundary. The system solves an inverse problem to find the capacitance distribution on the surface. In this paper, we assume that the conductive sheet used for capacitance coupling is a pure resistive material and that only electrically-grounded objects are considered as target objects. The following discussion is all under a single time frame, and all the variables are time-dependent.

**Figure 2:**
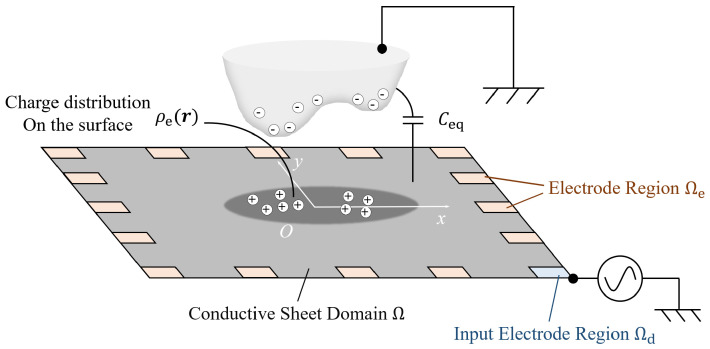
Explanation of proximity capacitance coupling and electrode position illustration (modified from the work in [21]).

As shown in Figure 2, when a grounded object exists above a conductive surface, capacitance $C_{\mathrm{eq}}$ appears between these two objects. If voltage $V_{\mathrm{in}}$ is applied at the boundary of the surface, free electrons will accumulate on the surface and the grounded object. The distribution of electrons is represented by $\rho_{e}\left(r\right)$ as the value of the charge density at point $r\in R^{2}$. We also define ϕ(r) as the potential value at the same point. As a result, capacitance density $\varsigma_{C}\left(r\right)$ can be defined on the surface as

(1) $$\varsigma_{C}\left(r\right)=\frac{\rho_{e}\left(r\right)}{\phi (r)}.$$

The shape and distance of the grounded object placed over the surface determine the capacitance density. Establishing the precise relation between the capacitance density and the distance distribution can be considerably difficult. However, the distance between the target object and the detecting sheet can be inferred by obtaining the capacitance density.

For the sensor design, *N* electrodes are attached to the sheet at the boundary, as shown in Figure 2. By inputting a voltage signal through one electrode and reading voltage data from other electrodes one at a time, we can obtain N−1 number of data for one input condition. After switching the input condition through all the boundary electrodes, we can obtain N(N−1) number of data for a single detection frame. Using those data to solve the inverse problem, we can obtain the capacitance distribution across the sheet. We can then estimate a proximity map of the grounded objects above the sensor.

### 2.2. Forward Problem

A reconstruction algorithm is used to solve the inverse problem of estimating $\varsigma_{C}$ using the potentials at the boundary electrodes for multiple input conditions. To define the inverse problem, we first consider the forward problem. Given the capacitance density $\varsigma_{C}\left(r\right)$ condition, the potential distribution on the sheet is obtained by solving a partial differential equation. As a thin sheet is utilized, the model is established on a 2D surface.

Considering the condition of free electrons at a point inside the conductive sheet domain Ω, the accumulation rate of its free electrons is equal to the inflow of the current to the point:

(2) $$\nabla \;\cdot \;j_{s}\left(r\right)+\frac{\partial \rho_{e}\left(r\right)}{\partial t}=0\;\mathrm{on}\;\Omega ,$$

where $j_{s}$ is the current density vector, $\rho_{e}$ is the amount of free electrons at that point, whose values are time variant, and *t* is time. The current density vector can be represented as in the following equation:

(3) $$j_{s}\left(r\right)=\sigma \left(r\right)E\left(r\right)=\sigma \left(r\right)\left(-\nabla \phi \left(r\right)\right),$$

where E(r) is the electric field, σ(r) is the conductivity of the homogeneous sheet, and ϕ(r) is the potential at the point. Equation (2) can then be rewritten as

(4) $$\nabla \;\cdot \;\left(-\sigma \left(r\right)\nabla \phi \left(r\right)\right)=\frac{\partial \rho_{e}\left(r\right)}{\partial t}.$$

Following the shunt model [22], the potential at the input electrode region $\Omega_{d}$ is treated as the boundary condition

(5) $$\phi \left(r\right)=V_{0}e^{j\omega t}\;\mathrm{on}\;\Omega_{d},$$

where $V_{0}$ is the voltage amplitude of the input sine signal and ω is its angular frequency. The potential at every point in the domain can then be represented as

(6) $$\phi \left(r\right)=V\left(r\right)e^{j(\omega t+\varphi (r))}\;\mathrm{on}\;\Omega ,$$

where V(r) is the amplitude and φ(r) is the phase. According to the previous definition of the capacitance density $\varsigma_{C}\left(r\right)$ in (1), the density of free electrons on position r is

(7) $$\rho_{e}\left(r\right)=\varsigma_{C}\left(r\right)V\left(r\right)e^{j(\omega t+\varphi (r))}.$$

A differential equation can then be derived from (4) and (7):

(8) $$\nabla \;\cdot \;\left(\sigma \left(r\right)\nabla \phi \left(r\right)\right)-j\omega \varsigma_{C}\left(r\right)\phi \left(r\right)=0.$$

Note that ϕ(r) is a complex number. Because the conductivity of the sheet does not change in our application, the variable in (8) is $\varsigma_{C}\left(r\right)$.

The finite element method (FEM) is used to find the potential distribution with (8). By solving the forward problem, the potentials at the electrodes can be calculated, and the output on electrode areas $\Omega_{e}$ can be represented as

(9) $$\phi \left(r\right)=V_{e}\left(r\right)e^{j(\omega t+\varphi_{e}\left(r\right))}\;\mathrm{on}\;\Omega_{e}.$$

### 2.3. Inverse Problem

A regularization-based imaging method [23] is utilized due to its fast reconstruction speed. Amplitude $V_{e}$ is used as our main measurement parameter, as it is easier for signal measurement. The basic idea of the dynamic imaging is linearizing the system around an initial density value vector $\varsigma_{0}$. After obtaining the Jacobian matrix J between the electrode potential amplitudes and the capacitance density, the differential amplitude vector δV can be approximately calculated using the following equation:

(10) δV≈Jδς+w

where δV is a vector comprising the difference of the current electrode amplitude readings and the original amplitudes, which are recorded before locating objects near the sensor; vector δς is the change of the capacitance density on each element; and vector w represents an error. The amplitude vector has N(N−1) elements, while the capacitance density vector has $N_{\mathrm{elem}}$ elements. In practice, we obtain the original amplitude data $V_{\mathrm{ori}}$ when there is no object in the detection range. We then subtract $V_{\mathrm{ori}}$ from the measured data $V_{\mathrm{mea}}$ to obtain the difference amplitude vector δV and do the reconstruction.

Through this method, a mapping between the electrode potential information δV and the capacitance density δς can be established in a linear relation, and an acceptable reconstruction speed is achieved.

#### 2.3.1. Jacobian Matrix

The Jacobian matrix is the derivative with respect to the capacitance density change. The matrix could be calculated numerically by perturbing the capacitance density of each element in the mesh by δς. In practice, a difference approximation for J is obtained by dividing δV by δς:

(11) $$J_{i,k}=\frac{\delta V_{i}}{\delta \varsigma_{k}};i=1,\ldots ,N\left(N-1\right);k=1,\ldots ,N_{\mathrm{elem}}$$

where *N* is the number of electrodes and $N_{\mathrm{elem}}$ is the number of elements in the detection area mesh.

#### 2.3.2. Regularization

As shown in (10), solving δς from δV is a highly ill-posed problem. Therefore, the Tikhonov regularization method is utilized to solve this problem. Lionheart et al. [24] stated that the formal solution of Tikhonov regularization is proposed as follows:

(12) $$\hat{\delta \varsigma }={\left(J^{T}J+\lambda^{2}Q\right)}^{-1}J^{T}\delta V$$

where λ is a hyperparameter that controls the amount of regularization and Q is a regularization matrix which, in our case, is an identity matrix I. By pre-calculating the matrix ${\left(J^{T}J+\lambda^{2}Q\right)}^{-1}J^{T}$, a fast speed reconstruction of the capacitance density can be achieved.

### 2.4. Proximity Mapping and Calibration

Different from the previous paper [21], for proximity mapping, we used the following equation to approximately fit the distance with the capacitance for objects possessing a flat bottom surface. According to the capacitance between two infinite size planar boards $C=\varepsilon_{0}S/d$, the capacitance density can be derived as $\varsigma =\varepsilon_{0}/d$ where *d* is the distance between the two boards and $\varepsilon_{0}$ is vacuum permittivity. We generated the following equation to approximate the proximity and capacitance density:

(13) $$d_{k}=b_{1}+\frac{b_{2}}{\delta \varsigma_{k}}$$

where $d_{k}$ is the normal distance of the object from the *k*-th element and $\delta \varsigma_{k}$ is the output from the solver on the *k*-th element. Parameters $b_{1}$ and $b_{2}$ can be fitted from the calibration experiment data. However, for a complex-shaped object, a specific function has to be considered to yield a better result in decoupling proximity and capacitance, which means that a separate calibration is needed for differently shaped objects.

## 3. Implementation

### 3.1. Sensor Construction

**Figure 3:**
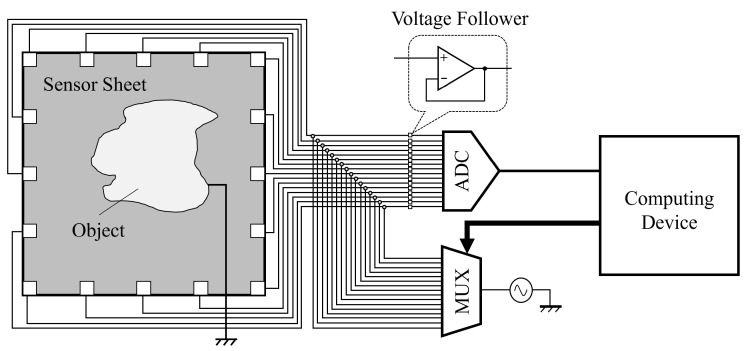
Outline of the sensor structure. Voltage followers are used to improve performance.

**Figure 4:**
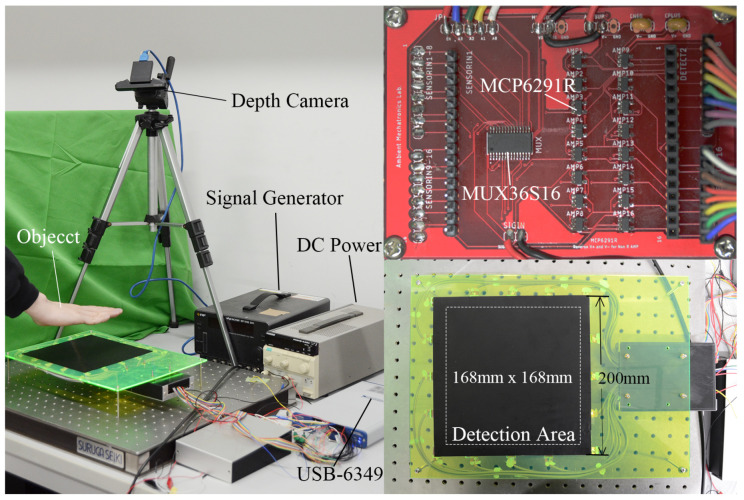
Sensor construction, circuit, and detection area.

The outline of the sensor is shown in Figure 3. A multiplexer switches the input signal on every electrode, and an AD converter (ADC) reads the voltage signal on every channel. Due to the large impedance in our system, voltage followers were implemented before every ADC to prevent the impedance of ADC affecting the sensor circuit. The multiplexer chosen was MUX36S16IPW, and the amplifier used for the voltage follower was MCP6291RT-E/MS. The circuit part is shown in Figure 4. For ADC, we used a USB-6349 Multifunction I/O Device (National Instrument), which could provide 500 kHz sampling speed for all the analog input channels. The control system was developed using LabView. For the sensor, a polyethylene-including carbon conductive sheet (ZC-86, Engineer Inc., Yokohama, Japan) with size of 200 mm×200 mm was used (see Figure 4). The surface resistance of the sheet is $5\times 10^{3}\;\Omega /\mathrm{Sq}$ at $20\;{}^{\circ }C$ temperature and 50% humidity, and its moisture permeability is $60\;g/m^{2}$. Sixteen copper square electrodes with a side length of 12 mm were arranged evenly on the perimeter and were connected to the sensor sheet with conductive tape.

A voltage with amplitude of 3 V and frequency of 20 kHz was used as the excitation signal. The signal generator used was WF-1946 (NF Corporation, Yokohama, Japan). This frequency was chosen because of the sampling speed for the ADC, and also because it generated the largest change in the electrode output amplitude with regard to the distance change of a hovering object. The channel selection on the multiplexer was controlled by USB-6349’s digital output channel. For the signal reading process, 500 sample points were recorded on every excitation condition. After reading one excitation condition, the controller changed the input condition. The switching speed of the controller was 1 kHz. For every excitation condition, the last 250 sample points were used to compute the amplitude. A hamming window was applied before applying fast Fourier transformation to erase the effect of phase difference. After recording all 16 excitation conditions, all data were sent to the inverse solver. The imaging frame rate for our prototype was 30 Hz.

### 3.2. Reconstruction Solver

The mesh used in the solver was generated by Altair Hypermesh software. In this paper, the size of the mesh was set to 200 mm×200 mm to match with the size of the real sensor. The domain was divided into 5000 isosceles right-angled triangle elements. The length of the cathetus on each triangle was 4 mm. Because we used a linear model to approximately solve the problem, the difference in size of the mesh elements affected the performance of the system greatly. Therefore, elements with the same size were applied across the whole domain. The side length of each square electrode was set to 12 mm. Python language was used to implement the forward FEM simulator and the inverse solver. For calculating the Jacobian matrix inside (10), a change of capacitance density $\delta \varsigma_{k}$ of $1\times 10^{-4}\;F/m^{2}$ was applied to one element. The corresponding electrode output amplitude $\delta V_{i}$ was obtained to construct the Jacobian matrix, as described in (11). The matrix was constructed by repeating this calculation on all 5000 elements. Choosing the hyperparameter λ in (12) considerably affected the reconstruction results. The optimal hyperparameter was chosen according to the best resolution method mentioned in [25], which was 203 in this paper. All the results mentioned in the succeeding paragraphs were all generated using the solver with optimized parameters.

**Figure 5:**
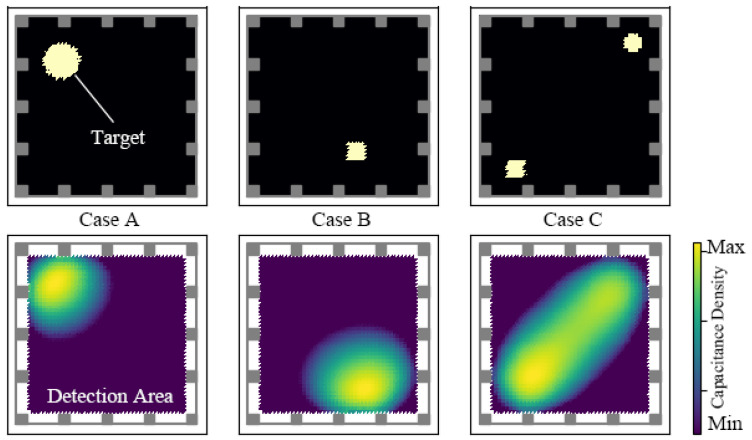
Reconstruction results of the algorithm. The first and second rows represent the ground truth and the reconstruction result, respectively. Inside ground truth image, the black area possesses a capacitance density of zero, while the yellow area is the ground truth target whose value is $1\times 10^{-9}\;F/m^{2}$.

The reconstruction solver was built after choosing the hyperparameters. Some simple examples were used to demonstrate the performance (see Figure 5). Reconstruction was applied in a detection area with a size of 168 mm×168 mm. A threshold process was applied to the result to show the pattern more clearly. We used 80% of the maximum value as the threshold value, and values smaller than the threshold are not shown in the result graph.

**Figure 6:**
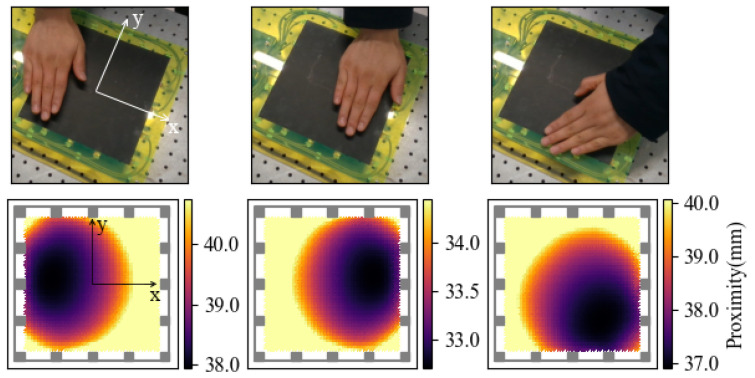
Proximity reconstruction results under three different hand-hovering conditions. A threshold of 80% value between the minimum and maximum proximity value is applied.

For parameters in (13), we used the calibration result of an 80 mm×80 mm object, which will be mentioned in Section 4 to fit $b_{1}$ and $b_{2}$. Figure 6 shows some measurement results of the constructed system.

## 4. Experiments

Two experiments were conducted to examine the performance of the sensor and show its potential applications. The first one was an evaluation experiment, and the other one was a hand-tracking demonstration.

### 4.1. Performance Evaluation

#### 4.1.1. Testing Device

To evaluate the performance of the sensor in proximity sensing, the proximity sensing performance (position accuracy on measuring objects) was investigated.

**Figure 7:**
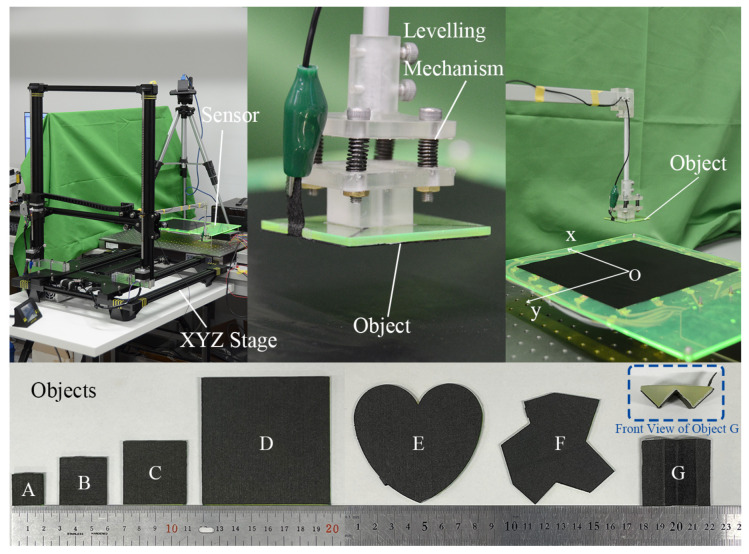
Experiment setup for performance evaluation.

In order to evaluate the accuracy of the 3D position sensing, an XYZ stage was prepared to control the object position. As shown in Figure 7, an ANYCUBIC Chiron 3D printer with 0.1 mm position accuracy was modified into an XYZ stage for moving the testing object. An aluminum frame was attached to the end of the moving component and the stage was calibrated so that its working space was parallel to the surface on which the sensor was placed. As shown in Figure 7, a leveling mechanism was attached between the object and the aluminum frame to compensate the tilt because of the extended part.

The objects used were made out of electron-conjugated conductive polymer sheet with a resistance of $2.5\times 10^{-2}\;\Omega /\mathrm{Sq}$ and acrylic boards which can be laser-cut freely and constructed into any shape. Four different sized square objects (objects A, B, C, and D in Figure 7) with side length of 20 mm, 30 mm, 40 mm, and 80 mm were chosen as the testing objects. Three other objects (objects E, F, and G in Figure 7) were chosen to demonstrate sensor’s capability of detecting randomly shaped objects and non-flat objects. These objects were grounded using a crocodile clipper attached to the slit that is extended from the bottom surfaces.

#### 4.1.2. Metrics

To evaluate the results, the center of position (CoP) was calculated according to the reconstruction result. The CoP was generated as the center of mass of the elements whose estimated capacitance density δς is larger than 90% of the maximum value. The capacitance density at CoP is the mean value of elements chosen to calculate CoP. In this work, position error (PE) was used as the main evaluation method at every height situation between the reconstructed CoP and the ground truth. For comparison between different objects, every object was hovered above the detection surface at ten different heights from 5 mm to 50 mm. At each height, the center of the object stayed at 25 different evenly distributed points inside the detection area (168 mm×168 mm). For randomly-shaped objects and non-flat objects, we tested these objects on three different heights: 10 mm, 30 mm, and 50 mm. The objects were also placed at 25 points same as the square objects.

To investigate the detection range of the sensor regarding the size of objects, the square objects were hovered above the center points at 20 different heights from 5 mm to 100 mm. The reconstructed capacitance density value at CoP was used to determine the detection range.

Before the experiment, every object was placed at 400 mm (where the object is far beyond the detection range) above the center point of the sensor to get the original voltage data $V_{\mathrm{ori}}$ for that object. We measured 50 datasets at every position and obtained the mean value of the output amplitude data, $V_{\mathrm{mea}}$. After gathering the amplitude output on every position, the difference between the gathered data and the original voltage data was used for the reconstruction.

#### 4.1.3. Results

**Figure 8:**
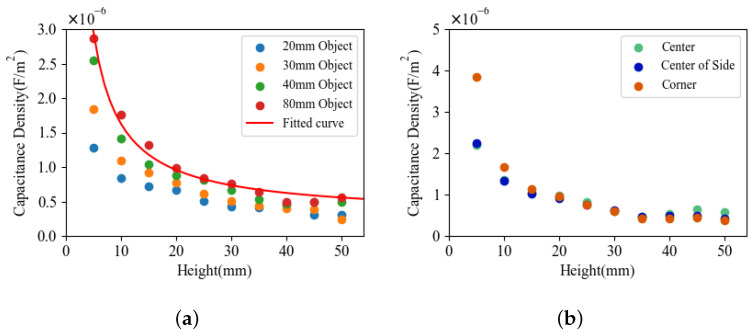
(**a**) Mean capacitance density value at center of position (CoP) reconstructed regarding distance using objects with different size. The red line shows the fitted curve of the 80 mm object. (**b**) Capacitance density value at CoP reconstructed regarding distance with different horizontal position using 80 mm object (Object D).

Figure 8a shows the mean capacitance densities at CoP at different heights for every object, and Table 1 summarizes their relative standard deviations (RSD). The result indicates that the capacitance density becomes smaller as the target object gets smaller.

However, the difference of the values between the 40 mm object and 80 mm object was not large, although the side length is two times larger. Because the electric field coupling is more complex on the perimeter of a flat object, larger objects posses more evenly distributed electric fields in the center. Due to this effect, the largest reconstructed value that represents the center value became similar on larger objects at the same height. We can utilize this property to implement some application on larger objects such as hand tracking. A simple flat object can be utilized to calibrate the sensor. The fitted parameters can then be used for hand-tracking experiments.

Note that the RSD value at 5 mm distance was much larger than at larger distances. The value difference is caused by the same effect described above. The electric fields is much more condensed at the perimeter of the sensor sheet. As shown in Figure 8b, at a smaller distance, the reconstructed value at the CoP is much larger on corner than the value at the center. As a result, the RSD value at 5 mm is significantly larger.

The last row of Table 1 demonstrates the detection range of every square object. Because of the noise, the proposed sensor performed poorly when the reconstructed capacitance value is smaller than $3\times 10^{-7}\;F/m^{2}$. As a result, we use this value as threshold of determining the maximum detection range for the objects. The height of the measurement point whose capacitance density value at CoP is the closest to the threshold was set as detection range. Due to stronger electrical coupling with larger objects, the detection range increases with the size of the object.

**Figure 9:**
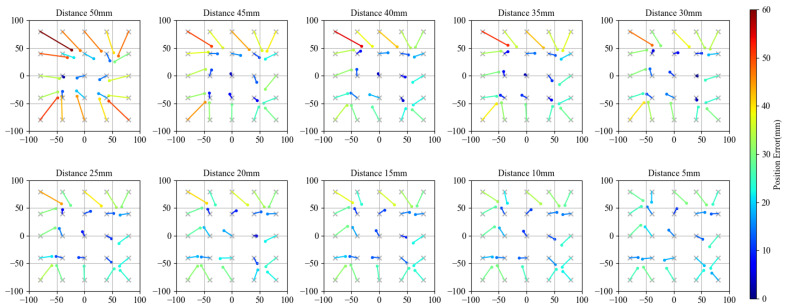
Reconstructed CoP compared with ground truth points in experiment with an 80 mm object. The points and gray crosses represent the reconstructed CoPs and the ground truths, respectively. Units of graphs is in millimeter mm.

Table 2 presents the PE value of the measurement results, and Figure 9 shows the CoP results for Object D (80 mm×80 mm). PE increased when height increased. For every object, the PE at each height value is similar, which means that the horizontal position accuracy on detecting a single object is similar regardless of the change in size.

Table 3 presents the Standard Deviation (STD) of the CoP coordinates of all samples at a single measurement on every height. The STD value represents the resolution of the sensor which varies with size and height of the object. The resolution is higher when objects are larger or object’s height is smaller.

**Figure 10:**
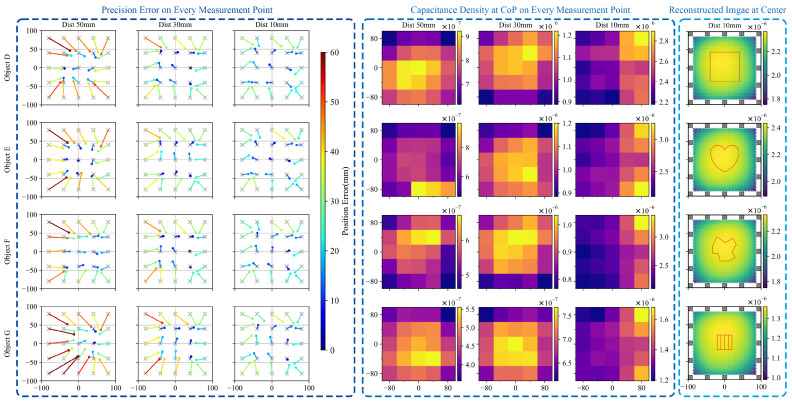
Sensor performance information on a reference object (object D) and three non-square object (objects E, F, and G). The first three columns present the position error of each measurement. The points and gray crosses represent the reconstructed CoPs and the ground truths, respectively. The columns in the middle demonstrate the reconstructed capacitance density value at CoP of each measurement. The reconstructed image is displayed in the last column. Inside the image, the object was hovering 10 mm above the central point of the sensor. Units of all graphs is in millimeter mm and units for capacitance density value is $F/{\mathrm{mm}}^{2}$.

The result of non-square objects (objects E, F, and G) is shown in Figure 10, which is similar to the result of squared objects with the same size. The results further indicates that the PE value is not concerned with the shape of the object. Furthermore, the data show that although with some larger error, the proposed sensor possesses the ability of detecting non-flat object.

### 4.2. Hand-Tracking Application

#### 4.2.1. Measuring Device

**Figure 11:**
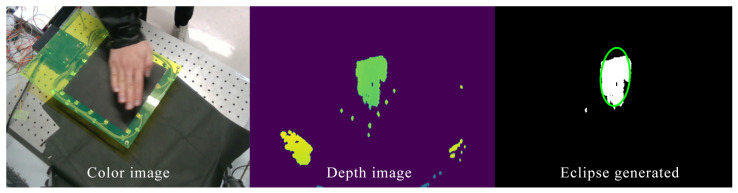
Hand position recognition process using depth camera.

To get the ground truth data of the hand, an Intel Realsense Development Kit SR300 depth camera was used to record the hand position simultaneously with our sensor (see Figure 11). A sleeve made of a material that is invisible to the depth camera was attached to the arm to restrict the visible area to the hand. First, the point cloud inside the camera coordinate is calculated from the depth image using the default information given by the data sheet. These points were then transformed from the camera coordinate $\left(x_{c},y_{c},z_{c}\right)$ to the sensor coordinate $\left(x_{s},y_{s},z_{s}\right)$. From the points in the sensor coordinate, the hand was then extracted as a set of points that satisfy ($|x_{s}\left|<300\;mm,\right|y_{s}|<300\;mm,z_{s}<300\;mm$). The contour of the hand inside the depth image was then generated using OpenCV library, as shown in Figure 11. The center position of the hand was determined by the eclipse covering the contour. The center of the oval was used as the ground truth value for the hand position. The camera captures the upper surface of the hand, whereas our sensor detects the lower surface. Therefore, after getting the center point of the hand’s upper surface $z_{\mathrm{up}}$, we estimated the height of the lower surface $z_{\mathrm{low}}$ using the relationship $z_{\mathrm{low}}=z_{\mathrm{up}}-15\;mm$.

To estimate the hand position with the proposed sensor, we used the CoP of a reconstruction image as *X* and *Y* axis’s position estimation of the hand. The capacitance density at CoP is used to estimate the distance of the hand from the sensor. The human subject did not touch any grounded object but weakly coupled with the ground. To accomplish the estimation, the sensor has to be calibrated. The result shown in Figure 9 indicates that the estimated positions shrink towards the center when the distance gets larger. The estimation of position is not correlated with the size of the object. As a result, a model was used to fit the estimated CoP for the ground truth to get a more accurate position. The relationship between the estimated CoP and ground truth is complicated; however, inside this paper, a linear model (14) was used to simplify calculation:

(14) $$\begin{array}{cc} \; & x^{\prime }=\left(c_{1}z+c_{2}\right)x_{s}\\ \; & y^{\prime }=\left(c_{1}z+c_{2}\right)y_{s}\\ \; & z^{\prime }=z_{s} \end{array}$$

where $c_{1}$ and $c_{2}$ were fitted from the calibration experiment using an 80 mm×80 mm object previously mentioned. The calibration took place at ten heights from 10 mm to 100 mm. For each height, 25 points in the horizontal plane, consistent with the previous experiment, were measured. In addition, (13) was used to fit the output capacitance density at CoP with the distance of the calibration object.

#### 4.2.2. Results

**Figure 12:**
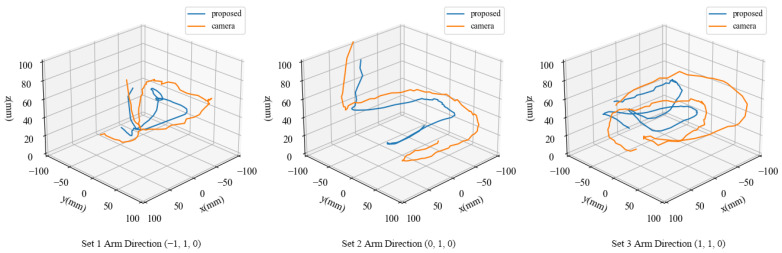
Comparison of hand trajectories between the sensor and depth camera. The blue and orange lines represent the sensor and the camera, respectively.

Three sets of experiment were carried out under three different hand and arm directions. Figure 12 shows the hand position estimation generated by the camera and the sensor, and Table 4 shows the position difference between the two methods in every set of experiment. Every data consists of 75 frames hand movement (around 2.5 s). The three different arm directions are (−1,1,0),(0,1,0),(1,1,0) from the hand center in the sensor base coordinate. The mean distance between the sensor estimation and the depth camera was approximately 15% of the sheet side length throughout the three sets of experiments. In most cases, the correlation coefficient between the proposed sensor and the camera system is larger than 0.9. Because the arm can also be detected by the sensor, the results tended to be biased towards the arm at a larger distance from the sensor sheet. Besides, the error between the linear model (14) and the actual relationship between estimated CoP and the ground truth might caused the gap between sensor estimation and camera results. In addition, as the detection area was limited, sensitivity at the perimeter was lower than that inside the detection area (168 mm×168 mm) bringing larger error in the result at perimeter areas.

### 4.3. Discussion

The proposed proximity imaging sensor yielded a prominent result in single object 3D position detection. Inside the performance evaluation experiment, the proposed sensor achieved horizontal errors of 10.0–19.8% of the sheet length and variations of the proximity at approximately 10%. For the hand-tracking task, the proposed system successfully estimated the hand position in 3D space. As shown in Table 4, the system reached a PE of 33.4–46.6 mm, which is smaller than half a hand-width. In most cases the correlation coefficient between the depth camera and the proposed system was larger than 0.9. Therefore, we conclude that the proposed sensor successfully estimated the 3D position information.

As shown in Table 2, the precision of positioning a single object was similar with differently sized objects. This means that using a larger sensor for the same object would not improve the positioning accuracy. However, the detection range might change with the shape of the sheet. Particularly, increasing the size of the sheet might help detect the same object at a larger distance.

Besides, the position accuracy of the proposed sensor can be improved. The contact impedance at the electrodes might affect the distribution of current inside the sensor material, resulting in distortion of the CoP from ground truth inside Figure 9. The error caused by contact impedance can be addressed by applying scaling function comparing with the simulation data [18]. Furthermore, for single object detection, a more precise fitting model substituting (14) can be used to increase position accuracy.

The temporal characteristic of the sensor is mainly concerned with the sampling speed of ADC, switching speed, and signal frequency. In this work, the imaging frame rate of the sensor was 30 Hz due to software limitations. With a more integrated software system, the theoretical frame rate could reach 60 Hz using the current hardware setup. On the other hand, the signal frequency is hard to increase because it affects the performance of the sensor, and higher frequency might also be difficult for portable ADC.

Although the experiment conducted herein exhibited the feasibility of the proposed sensor, some limitations must be acknowledged. First, it is difficult to find a universal function for relating proximity and capacitance density. Inside the proposed system, a function was hypothesized specifically for flat-bottom objects. However, for complex objects, this method is very approximate, restricting the precision of the sensor. In future works, we will first try to figure out how to discern the size of the object based on the acquired data.

Second, as the sensing area is directly exposed to the environment, environmental noise affects the performance of the proposed system. If the voltage change triggered by the object is smaller than noise, the object is undetectable. Currently, a simple signal processing was applied in order to exclude the effect of the phase change in obtaining the amplitude of the signal. In future works, the resolution of the sensor might increase by adapting more sophisticated signal processing techniques.

Last, multiple objects detection is difficult to realize with the current reconstruction algorithm. When two objects were hovering very close to the sensor (closer than 1 mm), the algorithm could distinguish two objects in the reconstructed image. However, distinguishing these objects was infeasible at a larger distance. Applying different Jacobian matrices for different heights or implementing different reconstruction approaches like the D-Bar method [26] and deep learning method [27,28,29,30] might help solve this issue in the future.

## 5. Conclusions

In this paper, we presented a proximity imaging sensor based on the tomographic approach using a low-cost conductive sheet. A novel theoretical model to image proximity using capacitance–proximity coupling was proposed to achieve the design of the sensor. A prototype sensor using the proposed model was implemented and tested. The position accuracy evaluation performed on the prototype revealed that the sensor reached a horizontal localization error rate of 10.0–15.8% at different height conditions inside the detection range. A hand-tracking application was carried out on the proposed sensor. Compared with the depth camera system, the proposed system achieved a position error of 33.38–46.66 mm, proving its feasibility. Therefore, the proposed system is expected to be used in various robotic applications, such as teleoperation, input interface, and interaction between human, machine, and environments.

## Figures and Tables

**Table 1:** Relative standard deviation (RSD) of capacitance density value at CoP on every height. The last row presents detection range of every square object. The values are height of the measurement point whose reconstructed capacitance density is closest to $3\times 10^{-7}\;F/{\mathrm{mm}}^{2}$.

	Object A	Object B	Object C	Object D
	20 mm	30 mm	40 mm	80 mm
Distance (mm)	RSD (%)	RSD (%)	RSD (%)	RSD (%)
5	20.5	21.9	22.2	27.5
10	6.0	6.5	7.9	8.3
15	4.9	4.2	7.9	6.4
20	9.1	7.2	7.3	8.9
25	6.3	5.5	5.2	8.5
30	5.9	6.5	6.4	8.3
35	8.1	6.2	9.8	8.6
40	11.5	10.2	11.7	7.8
45	9.1	9.3	12.0	12.6
50	10.2	10.9	10.9	10.9
**Detection Range** (mm)	50	60	70	90

**Table 2:** PE data for experimental data and simulation data.

	Object A		Object B		Object C		Object D	
	20 mm × 20 mm		30 mm × 30 mm		40 mm × 40 mm		80 mm × 80 mm	
Distance (mm)	Mean (mm)	STD (mm)	Mean (mm)	STD (mm)	Mean (mm)	STD (mm)	Mean (mm)	STD (mm)
5	21.518	8.806	20.806	7.316	19.935	7.644	20.725	6.860
10	22.241	8.681	22.676	7.769	21.999	10.089	21.414	8.833
15	24.601	11.214	22.909	8.696	26.662	14.314	22.067	10.144
20	28.592	14.277	21.983	9.313	24.732	9.994	22.504	10.675
25	28.030	12.690	25.554	12.157	23.756	11.524	22.815	11.779
30	27.743	13.933	26.624	13.009	25.251	13.458	24.401	13.887
35	30.909	15.684	26.621	12.205	31.335	15.177	25.189	14.571
40	37.804	19.256	26.554	11.726	29.406	13.474	24.721	13.829
45	34.174	15.813	39.648	19.574	29.178	16.170	27.489	14.647
50	38.869	17.132	39.191	21.504	30.349	16.173	36.275	16.522

**Table 3:** Standard deviation (STD) of the CoP coordinates from all samples (50 frames × 25 points) at one measurement point on every height. The STD value of a single measurement point indicates the resolution of proposed sensor at the certain height.

	Object A		Object B		Object C		Object D	
	20 mm × 20 mm		30 mm × 30 mm		40 mm × 40 mm		80 mm × 80 mm	
Distance (mm)	x Axis (mm)	y Axis (mm)	x Axis (mm)	y Axis (mm)	x Axis (mm)	y Axis (mm)	x Axis (mm)	y Axis (mm)
5	0.826	0.915	0.503	0.555	0.331	0.364	0.676	0.845
10	1.282	1.388	0.731	0.741	0.583	0.648	0.827	1.025
15	1.558	1.503	1.601	1.192	0.765	0.861	1.187	1.461
20	1.805	1.759	1.424	1.737	1.829	1.917	1.339	1.544
25	2.328	2.400	1.481	1.573	1.877	1.483	1.654	1.838
30	3.030	2.701	1.669	1.993	1.188	1.453	1.560	1.809
35	3.545	2.907	2.221	2.919	1.735	1.878	2.227	2.650
40	2.995	2.540	2.544	2.524	2.266	2.406	2.268	2.650
45	3.770	4.120	2.903	2.679	3.370	2.943	2.597	2.871
50	4.217	3.776	3.052	2.842	2.434	2.550	2.528	2.460

**Table 4:** Position difference between camera estimation and sensor estimation.

Experiment Set	Set 1	Set 2	Set 3
Arm Direction	(−1,1,0)	(0,1,0)	(1,1,0)
Mean Distance (mm)	46.66	33.38	45.14
Mean X Difference (mm)	32.45	18.7	21.7
Mean Y Difference (mm)	10.62	21.4	23.2
Mean Z Difference (mm)	6.31	9.5	10.4
X Correlation Coefficient	0.905	0.954	0.892
Y Correlation Coefficient	0.949	0.981	0.907
Z Correlation Coefficient	0.851	0.933	0.725

## Data Availability

Data can be requested by direct email contacts to the author.
